# Effect of Hydroalcoholic Ginger Extract on Brain HMG-CoA Reductase and CYP46A1 Levels in Streptozotocin-induced Diabetic Rats

**Published:** 2019

**Authors:** Shirin Azizidoost, Zahra Nazeri, Asma Mohammadi, Ghorban Mohammadzadeh, Maryam Cheraghzadeh, Alireza Jafari, Alireza Kheirollah

**Affiliations:** 1. Department of Biochemistry, Faculty of Medicine, Ahvaz Jundishapur University of Medical Sciences, Ahvaz, Iran; 2. Department of Biochemistry, Abadan University of Medical Sciences, Abadan, Iran; 3. Hyperlipidemia Research Center, Department of Clinical Biochemistry, Faculty of Medicine, Ahvaz Jundishapur University of Medical Sciences, Ahvaz, Iran; 4. Department of Biochemistry, Cellular and Molecular Research Center, Ahvaz Jundishapur University of Medical Sciences, Ahvaz, Iran

**Keywords:** Brain, Cholesterol 24-hydroxylase, Diabetes mellitus, Ginger, Hydroxymethylglutaryl-CoA reductases

## Abstract

**Background:**

Patients with diabetes present with lipid disorders, including hypercholesterolemia, which can be a high-risk factor for atherosclerosis. Recently, increasing interest has been focused on anti-lipidemic function of herbal medicines, especially *Zingiber officinale* (known as ginger), in diabetes. However, the mechanism underlying the effect of ginger on some players involved in cholesterol homeostasis of Central Nervous System (CNS) among diabetic patients remains unclear. To our knowledge, this is the first study to investigate the effect of ginger on brain regulation of Hydroxymethylglutaryl-CoA Reductase (HMG-CoA reductase) and Cholesterol 24-hydroxylase (CYP46A1), which provides a rational model for understanding brain dyslipidemia mechanisms associated with diabetes.

**Methods:**

Brains of rats were isolated from four groups: control, non-treated diabetic, and treated diabetic groups receiving 200 or 400 *mg/kg* of hydroalcoholic extracts of ginger for eight weeks. HMG-CoA reductase and CYP46A1 levels in brain homogenates were determined by western-blot technique.

**Results:**

Ginger root extract caused a significant decrease in HMG-CoA reductase and an increase in CYP46A1 levels in treated diabetic groups compared to diabetic control. In comparison to diabetic group, these effects were more remarkable with 400 *mg/kg* concentration of ginger extract.

**Conclusion:**

The findings showed that ginger extract has a regulatory effect on proteins involved in cholesterol homeostasis in CNS by a significant down- and up-regulation of HMG-CoA reductase and CYP46A1 levels, respectively. It can be suggested that adding ginger to daily diet of diabetic patients has useful effects and may ameliorate diabetes complications.

## Introduction

Diabetes mellitus is a chronic endocrine disorder characterized by increased blood glucose levels as a consequence of impaired insulin function or secretion 1. It should be noted that the prevalence of diabetes is increasing worldwide. In addition to hyperglycemia, hypercholesterolemia is also attributed to diabetes. In this respect, atherosclerosis after dyslipidemia is the most prevalent complication of diabetes mellitus 2. The association between diabetes and age-related dementias like Alzheimer’s disease has been a matter of debate 3. In this regard, hypercholesterolemia caused by diabetes leads to elevated brain amyloid-beta levels as well as severe learning and memory impairments 4. So, it is said that type II diabetes is a risk factor for vascular dementia 5,6. Since increased cholesterol synthesis concomitant with impaired cholesterol absorption is reported in diabetes 7,8, alteration of cholesterol homeostasis in brain can help understand its dyslipidemia mechanism in diabetes. Furthermore, over 25% of the body’s cholesterol pool is located in the Central Nervous System (CNS) and brain cells are segregated from lipoproteins in the systemic circulation by the blood-brain barrier 9. So, cholesterol homeostasis in the brain is unique and dependent on its own de novo synthesis and degradation with a specific extracellular lipid transport system. It is of note that brain cholesterol metabolism is also involved in several physiological function in the CNS including myelin production 10. Moreover, SREBP-Cleavage Activating Protein (SCAP), which is a chaperone of Sterol Regulatory Element-Binding Protein 2 (SREBP2), inhibits the overload of intracellular cholesterol whenever cells preserve adequate cholesterol 11. It has been proved that the up-regulation of SREBP-2 expression as a result of SCAP over-expression elevates cholesterol uptake, which results in cholesterol accumulation in diabetic rats 12,13. So, regarding previous findings about memory loss of hypercholesterolemia caused by diabetes, it should be elucidated that diabetes complications affect other cholesterol metabolism-related gene in the brain and also lead to a more accurate understanding of alterations of cholesterol metabolism in diabetes. It has been shown that the variation of both plasma and Β-cell islet cholesterol homeostasis may correlate to the pathogenesis of diabetes 14. Lack of ATP binding cassette subfamily A member 1 (ABCA1), which is one of the main players of cholesterol metabolism modulating cholesterol efflux, leads to overload of cholesterol in B-cell islet and impaired insulin release 15.

There is increasing interest in the application of herbal medicines against human disorders worldwide. *Zingiber officinale (Z. officinale)*, also known as ginger, is amongst important herbal medicines possessing anti-diabetic properties, the antihyperglycemic effects of which have been indicated in several studies 16. Besides *in vivo* studies, findings from clinical trials showed the protective effect of ginger extract in the reduction of blood glucose levels 17. However, the effect of hydroalcoholic extract of ginger on the alteration of some enzymes involved in brain cholesterol homeostasis, including 3-hydroxy-3-methylglutaryl-coenzyme A reductase (HM G-CoA reductase) and cytochrome P450 family 46 subfamily A member 1 (CYP46A1), is poorly understood in diabetes.

It is of interest to check the effect of ginger extract on brain cholesterol homeostasis in a streptozotocin-induced diabetic rat model given the increased levels of major components of lipid profile in diabetes mellitus.

## Materials and Methods

### Materials

The dried root of ginger (*Z. officinale*) was purchased from Goldaru Isfahan Pharmaceutical Company, Isfahan, Iran. Mouse anti-β-actin antibody was obtained from Sigma Aldrich, PVDF was obtained from Roche Applied Science, and mouse anti-CYP 46A1 was purchased from Santa Cruz. Mouse Anti-HMG-CoA reductase was kindly donated by Professor TY Chang, Geisel School of Medicine at Dartmouth, Hanover, NH, USA. All other chemicals were purchased from Merck chemicals, Germany.

### Animal model

40 male Wistar rats weighting 200–250 *gr* (8–10 weeks old), which were obtained from research center and experimental animal house of Jundishapur University of medical sciences, Ahvaz, Iran were selected for experimental tests in this study. Before all analytical tests, animals were acclimatized under standard ad libitum conditions for 3 days.

### Extract preparation

The dried roots of ginger (*Z. officinale*) were purchased from Goldaru Herbal Pharmaceutical Company, Isfahan, Iran. The voucher samples were preserved for reference in the Biochemistry Department of Medical School, Ahvaz Jundishapur University of Medical Sciences. Ginger extraction was done by filtration. Briefly, 200 *gr* of ginger roots was crushed with a blender and then soaked in 1400 *ml* of 70% methyl alcohol for 3 days. After filtration of homogenized mixture using Whatman filter paper No.40, the filtrate was placed under vacuum at 50°C to evaporate methanol. Finally, 25 *gr* crystallized extract was obtained.

### Induction of diabetes by streptozotocin

Induction of diabetes was performed by intravenous administration of 40 *mg/kg* streptozotocin (STZ) dissolved in cold 0.1 *M* citrate buffer (pH=4.5). After 3 days of STZ administration, tail vein blood was taken to measure fasting blood glucose with a glucometer. Diabetes was verified according to plasma glucose concentrations higher than 350 *mg/dl*.

### Experimental animal groups

40 rats were randomly divided into 4 groups. Group 1 consisted of 10 rats as non-diabetic control, and each rat received 1.5 *ml/kg* distilled water daily by gavage. Also, two weeks after induction of diabetes, diabetic rats were randomly divided into 3 experimental animal groups (10 rats each) as follows: Group 2 as non-treated diabetic group in which each rat received 1.5 *ml/kg* distilled water daily by gavage, Group 3 as the diabetic group that received 200 *mg/kg* hydroalcoholic extract of ginger dissolved in 1.5 *ml/kg* distilled water daily by gavage, and Group 4 as the diabetic group that received 400 *mg/kg* hydroalcoholic extract of ginger dissolved in 1.5 *ml/kg* distilled water daily by gavage. The treatment lasted for eight weeks, and all experiments were done after two weeks of STZ administration.

### Tissue preparation and western blotting

Mice from experimental groups were anesthetized and their brain homogenates were prepared as follows. After dissection, the brain was washed with dPBS and homogenized in ice-cold RIPA buffer with protease inhibitor cocktail using sonication. An equal amount of proteins in brain homogenates was subjected to 8% sodium dodecylsulfate-polyacrylamide gel electrophoresis (SDS-PAGE) and transferred to a polyvinylidene fluoride membrane (Roche). After blocking with blocking buffer containing 5% skim milk in TBST, the membrane was incubated overnight with specific primary antibodies for HMG-CoA reductase (1:5000 dilution, Abcam), CYP46A1 (1:500 dilution, Santacruz), and Β-actin (1:5000 dilution, Sigma). After washing the membrane with TBST, it was incubated for 1.5 *hr* with specific goat anti-mouse (1:4000 dilution, Sigma) secondary antibodies for HMG-CoA reductase, CYP 46A1, and Β-actin. After chemiluminescence reaction, the bands were visualized using ChemiDoc™ (Bio-Rad).

### Statistical analysis

Statistical analysis was performed with SPSS (Version 18) Software. Descriptive statistics presented data as mean±SD. Analysis of Variance (ANOVA) was used to check significant differences between groups in western blotting analysis. To quantify the difference between protein levels, the data were analyzed using ImageJ software. The quantification reflects the relative amounts as a ratio of each protein band relative to the lane’s loading B-actin as control. For all statistical analysis, p<0.05 was considered as the significance level.

## Results

### Effect of hydroalcoholic Z. officinale extract on HMG-CoA reductase levels in brain

**Figure 1.**
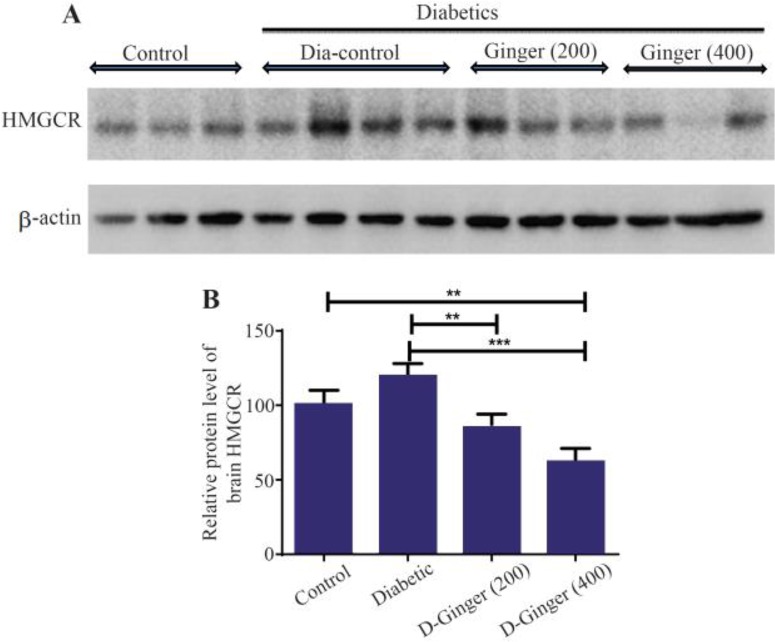
Effect of hydroalcoholic ginger extract on the protein levels of HMG-CoA reductase in the brain. A) Representative immune-blotting showing specific bands for HMG-CoA reductase. β-actin is used as an internal control. B) Graphic presentation of data obtained from western blot analysis. Each bar shows mean±SD. Significant difference from diabetic group was shown as p<0.05. From left to right, lanes 1, 2, and 3 are related to control; lanes 4, 5, 6, and 7 are related to diabetic group (DM) without any treatments; lanes 8, 9, and 10 are related to diabetic group receiving 200 *mg/kg* ginger extract; and lanes 11, 12, and 13 are related to diabetic group receiving 400 *mg/kg* ginger extract, respectively.

Brain homogenate was prepared using sonication in ice-cold RIPA buffer with protease inhibitor cocktail. The effect of ginger extract on the protein levels of HMG-CoA reductase in brain homogenate was analyzed by SDS-PAGE and western blotting. Although the level of HMG-CoA reductase in diabetic rats treated with 200 *mg/kg* concentration of ginger extract was approximately equal to the control group, treatment of streptozotocin-induced diabetic rats with hydroalcoholic extract of ginger for eight weeks caused reduced HMG-CoA reductase compared to diabetic group in the brain. This effect was more pronounced with 400 *mg/kg* of ginger extract (Figure 1), indicating the effect of ginger on rate-controlling enzyme of mevalonate pathway, which is the metabolic pathway producing cholesterol and other isoprenoids.

### Effect of hydroalcoholic Z. officinale extract on brain CYP46A1 levels

To check the effect of ginger on brain CYP46A1 content, the brain homogenate was subjected to SDS-PAGE and western blotting. CYP46A1 level in diabetic non-treated rats decreased compared to control group in the brain but the influence of ginger treatment increased CYP46A1 levels compared to diabetic rats since it reached to control level with 400 *mg/kg* concentration of ginger extract. Among the treated diabetic groups, increased level of CYP46A1 in diabetic group receiving 200 *mg/kg* of ginger was significant in comparison to the control group and level of CYP46A1 in diabetic ones receiving 400 *mg/kg* of ginger was almost as normal as the control group.

## Discussion

In this study, the effect of hydroalcoholic *Z. officinale* extract was investigated on brain HMG-CoA reductase and CYP46A1 levels in streptozotocin-induced diabetic rats. Although the changes in levels of enzymes involved in lipid metabolism due to diabetes have been a matter of debate, our data demonstrated the up-regulation of HMG-CoA reductase in diabetes, and the use of ginger extract caused a modulation in HMG-CoA reductase level and could return it back to the basal level of control group. Although CYP46A1 protein level decreased in diabetes, its level is increased by ginger extract treatment; with 400 *mg/kg* of ginger extract, it reached to control group’s level, which could reduce the cholesterol accumulation and it has beneficial effects on cholesterol hemostasis in the brain.

Dyslipidemia is among the most important complications of diabetes, which predisposes people to cardiovascular diseases 18. A significant rise of triglyceride and cholesterol along with decrease of HDL cholesterol can be attributed to endogenous alterations of lipid homeostasis caused by diabetes. Since the neural cells in CNS are segregated from lipoproteins in the systemic circulation by the Blood-Brain Barrier (BBB) 19, here the focus was on the level of two enzymes involved in brain cholesterol hemostasis in a diabetic rat model, HMG-CoA reductase and CYP46A1, and it was found that the administration of ginger extract significantly reduced HMG-CoA reductase protein level in comparison to control or diabetic group. This reduction was more pronounced in diabetic group receiving 400 *mg/kg* of ginger. By contrast, up-regulation of CYP 46A1 in diabetic rats treated with ginger extract in comparison to diabetes groups verified its role in stimulating the elimination of excess cholesterol from the brain.

**Figure 2.**
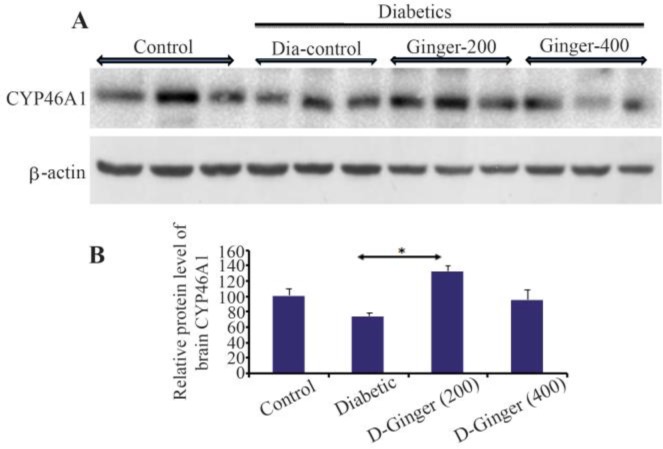
Effect of hydro alcoholic extract of ginger on the protein levels of CYP46A1 in the brain. A) Representative immune-blotting showing specific bands for CYP46A1. β-actin is used as an internal control. B) Graphic presentation of data obtained from western blot analysis. Each bar shows mean±SD. From left to right, lanes 1, 2, and 3 are related to control; lanes 4, 5, and 6 are related to diabetic group (DM) without any treatments; lanes 7,8, and 9 are related to diabetic group receiving 200 *mg/kg* ginger extract; and lanes 10, 11, and 12 are related to diabetic group receiving 400 *mg/kg* ginger extract, respectively.

It is well documented that CYP46A1, which is mainly expressed in the brain, enzymatically produces 24S-hydroxycholesterol (24S-HC) from cholesterol to enhance cholesterol turnover, eliminate surplus cholesterol, and regulate cholesterol synthesis 20. It is believed that there is a balance between HMG-CoA reductase and CYP46A1 protein levels in CNS and along with HMG-CoA reductase increase, the protein level of CYP46A1 will be increased to keep the cholesterol level in a steady-state condition. Our data showed that this harmony between HMG-CoA reductase and CYP-46A1 is disrupted in diabetes; however, ginger extract can correct this disturbing effect (Figure 2). As it is shown in the diabetic group receiving 200 *mg/kg* of ginger extract, CYP46A1 protein level is still up regulated and as HMG-CoA reductase protein level decreased by 400 *mg/kg* of ginger extract, CYP46A1 is reduced and almost reached to the control group’s level suggesting a parallel balance between cholesterol synthesis and cholesterol elimination under steady-state conditions.

If CYP46A1 level is increased by ginger extract, it may regulate brain cholesterol content through two mechanisms. First, CYP46A1 enhances the hydroxylation of cholesterol to generate 24S-HC, which is soluble and can pass BBB to be excreted into circulation 21. Second, it increases the generation of 24S-HC, which is perhaps attributed to the inhibition of brain cellular cholesterol biosynthesis by down-regulation of HMG-CoA reductase *via* blocking SREBPs, the main controllers of cholesterol biosynthesis after administration of ginger extract 22,23. Reduced brain cholesterol biosynthesis may coincide with the fact that increased expression of CYP46A1 after consumption of ginger cooperates with HMG-CoA reductase in order to prevent cholesterol accumulation in the brain.

Here, the effect of hydroalcoholic ginger extract on main players of proteins involved in cholesterol homeostasis in CNS was shown, which could be in line with the lipid-lowering activity of ginger extract in circulation reported by others who showed a significant reduction of serum lipid profile, including cholesterol and triglyceride, after ginger administration to diabetic rats 24.

## Conclusion

Taken together, our findings revealed a possible mechanism for the cholesterol-lowering effect of ginger in a diabetic animal model. Our data regarding the regulatory effect of ginger on players involved in brain cholesterol homeostasis along with other reports 25 showing the reduction of brain oxidative damage, apoptosis, and inflammation in streptozotocin-induced diabetic rats revealed the neuroprotective effect of ginger against structural brain alteration associated with diabetes.

## References

[1] Mustafa SS Eid NI Jafri S El-Latif HAA Ahmed HM Insulinotropic effect of aqueous ginger extract and aqueous garlic extract on the isolated perfused pancreas of streptozotocin induced diabetic rats. Pakistan J Zool 2007;39(5):279–284.

[2] Al-Azhary DB Ginger enhances antioxidant activity and attenuates atherogenesis in diabetic cholesterolfed rats. Aust J Basic Appl Sci 2011;5(12):2150–2158.

[3] Luchsinger JA Tang MX Stern Y Shea S Mayeux R Diabetes mellitus and risk of Alzheimer's disease and dementia with stroke in a multiethnic cohort. Am J Epidemiol 2001;154(7):635–641. 11581097 10.1093/aje/154.7.635

[4] Martins I Hone E Foster J Sünram-Lea S Gnjec A Fuller S Apolipoprotein E, cholesterol metabolism, diabetes, and the convergence of risk factors for Alzheimer's disease and cardiovascular disease. Mol Psychiatry 2006;11(8):721–736. 16786033 10.1038/sj.mp.4001854

[5] Bruce DG Casey GP Grange V Clarnette RC Almeida OP Foster JK Cognitive impairment, physical disability and depressive symptoms in older diabetic patients: the Fremantle Cognition in Diabetes Study. Diabetes Res Clin Pract 2003;61(1):59–67. 12849924 10.1016/s0168-8227(03)00084-6

[6] Stewart R Liolitsa D Type 2 diabetes mellitus, cognitive impairment and dementia. Diabet Med 1999;16(2):93–112. 10229302 10.1046/j.1464-5491.1999.00027.x

[7] Pihlajamäki J Gylling H Miettinen TA Laakso M Insulin resistance is associated with increased cholesterol synthesis and decreased cholesterol absorption in normo-glycemic men. J Lipid Res 2004;45(3):507–512. 14657199 10.1194/jlr.M300368-JLR200

[8] Simonen PP Gylling HK Miettinen TA Diabetes contributes to cholesterol metabolism regardless of obesity. Diabet Care 2002;25(9):1511–1515. 10.2337/diacare.25.9.1511 12196419

[9] Suzuki R Lee K Jing E Biddinger SB McDonald JG Montine TJ Diabetes and insulin in regulation of brain cholesterol metabolism. Cell Metab 2010;12(6): 567–579. 21109190 10.1016/j.cmet.2010.11.006 PMC3205997

[10] Dietschy JM Turley SD Thematic review series: brain Lipids. Cholesterol metabolism in the central nervous system during early development and in the mature animal.. J Lipid Res 2004;45(8):1375–1397. 15254070 10.1194/jlr.R400004-JLR200

[11] Sun H Yuan Y Sun ZL Cholesterol contributes to diabetic nephropathy through SCAP-SREBP-2 pathway. Int J Endocrinol 2013;2013. 10.1155/2013/592576 PMC3863482 24369464

[12] Wang Z Jiang T Li J Proctor G McManaman JL Lucia S Regulation of renal lipid metabolism, lipid accumulation, and glomerulosclerosis in FVBdb/db mice with type 2 diabetes. Diabetes 2005;54(8):2328–2335. 16046298 10.2337/diabetes.54.8.2328

[13] Yuan Y Zhao L Chen Y Moorhead JF Varghese Z Powis SH Advanced glycation end products (AGEs) increase human mesangial foam cell formation by increasing Golgi SCAP glycosylation in vitro. Am J Physiol Renal Physiol 2011;301(1):F236–F243. 21511699 10.1152/ajprenal.00646.2010

[14] Brunham LR Kruit JK Verchere CB Hayden MR Cholesterol in islet dysfunction and type 2 diabetes. J Clin Invest 2008;118(2):403–408. 18246189 10.1172/JCI33296 PMC2214697

[15] Brunham LR Kruit JK Pape TD Timmins JM Reuwer AQ Vasanji Z β-cell ABCA1 influences insulin secretion, glucose homeostasis and response to thiazoli-dinedione treatment. Nat Med 2007;13(3):340–347. 17322896 10.1038/nm1546

[16] Li Y Tran VH Duke CC Roufogalis BD Preventive and protective properties of Zingiber officinale (ginger) in diabetes mellitus, diabetic complications, and associated lipid and other metabolic disorders: a brief review. Evid Based Complement Alternat Med 2012;2012:516870. 23243452 10.1155/2012/516870 PMC3519348

[17] Ojewole JA Analgesic, antiinflammatory and hypoglycaemic effects of ethanol extract of Zingiber officinale (Roscoe) rhizomes (Zingiberaceae) in mice and rats. Phytother Res 2006;20(9):764–772. 16807883 10.1002/ptr.1952

[18] Singh Rajput DP Shah JY Singh P Jain S Evaluation of dyslipidemia in type 2 diabetes mellitus. Asian J Med Sci 2015;6(6):16–19.

[19] Robert J Cheng WH Hayat A Ward-Able T Wellington CL High-density lipoproteins at the interface between central nervous system and plasma lipoprotein metabolism. Clin Lipidol 2017;10(1):69–81.

[20] Djelti F Braudeau J Hudry E Dhenain M Varin J Bieche I CYP46A1 inhibition, brain cholesterol accumulation and neurodegeneration pave the way for Alzheimer’s disease. Brain 2015;138(8):2383–2398. 26141492 10.1093/brain/awv166

[21] Boussicault L Alves S Lamazière A Planques A Heck N Moumné L CYP46A1, the rate-limiting enzyme for cholesterol degradation, is neuroprotective in Huntington’s disease. Brain 2016;139(3):953–970. 26912634 10.1093/brain/awv384 PMC4766376

[22] DeBose-Boyd RA Feedback regulation of cholesterol synthesis: sterol-accelerated ubiquitination and degradation of HMG CoA reductase. Cell Res 2008;18(6):609–621. 18504457 10.1038/cr.2008.61 PMC2742364

[23] Wang Y Muneton S Sjövall J Jovanovic JN Griffiths WJ The effect of 24S-hydroxycholesterol on cholesterol homeostasis in neurons: quantitative changes to the cortical neuron proteome. J Proteome Res 2008;7(4):1606–1614. 18303831 10.1021/pr7006076 PMC2374888

[24] Elshater A Salman MM Moussa MM Effect of ginger extract consumption on levels of blood glucose, lipid profile and kidney functions in alloxan induced-diabetic rats. Egypt Acad J Biolog Sci 2009;2(1):153–162.

[25] El-Akabawy G El-Kholy W Neuroprotective effect of ginger in the brain of streptozotocin-induced diabetic rats. Ann Anat 2014;196(2–3):119–128. 24680376 10.1016/j.aanat.2014.01.003

